# 11β-HSD1 contributes to age-related metabolic decline in male mice

**DOI:** 10.1530/JOE-22-0169

**Published:** 2022-10-07

**Authors:** Stuart A Morgan, Laura L Gathercole, Zaki K Hassan-Smith, Jeremy Tomlinson, Paul M Stewart, Gareth G Lavery

**Affiliations:** 1 Institute of Metabolism & Systems Research, University of Birmingham, Birmingham, UK; 2 Department of Biosciences, Nottingham Trent University, Nottingham, UK; 3 Department of Biological & Medical Sciences, Oxford Brooks University, Oxford, UK; 4 Radcliffe Department of Medicine, University of Oxford, Oxford, UK; 5 NEXUS, Discovery Way, University of Leeds, Leeds, UK

**Keywords:** glucocorticoids, 11β-HSD1, ageing, insulin resistance, obesity, Cushing’s syndrome

## Abstract

The aged phenotype shares several metabolic similarities with that of circulatory glucocorticoid excess (Cushing’s syndrome), including type 2 diabetes, obesity, hypertension, and myopathy. We hypothesise that local tissue generation of glucocorticoids by 11β-hydroxysteroid dehydrogenase type 1 (11β-HSD1), which converts 11-dehydrocorticosterone to active corticosterone in rodents (corticosterone to cortisol in man), plays a role in driving age-related chronic disease. In this study, we have examined the impact of ageing on glucocorticoid metabolism, insulin tolerance, adiposity, muscle strength, and blood pressure in both wildtype (WT) and transgenic male mice with a global deletion of 11β-HSD1 (11β-HSD1^−/−^) following 4 months high-fat feeding. We found that high fat-fed 11β-HSD1^−/−^ mice were protected from age-related glucose intolerance and hyperinsulinemia when compared to age/diet-matched WTs. By contrast, aged 11β-HSD1^−/−^ mice were not protected from the onset of sarcopenia observed in the aged WTs. Young 11β-HSD1^−/−^ mice were partially protected from diet-induced obesity; however, this partial protection was lost with age. Despite greater overall obesity, the aged 11β-HSD1^−/−^ animals stored fat in more metabolically safer adipose depots as compared to the aged WTs. Serum analysis revealed both WT and 11β-HSD1^−/−^ mice had an age-related increase in morning corticosterone. Surprisingly, 11β-HSD1 oxo-reductase activity in the liver and skeletal muscle was unchanged with age in WT mice and decreased in gonadal adipose tissue. These data suggest that deletion of 11β-HSD1 in high fat-fed, but not chow-fed, male mice protects from age-related insulin resistance and supports a metabolically favourable fat distribution.

## Introduction

People are now living longer than ever before. Globally, the proportion of people aged 60 years or over has increased from 9.2% in 1990 to 11.7% in 2013 and is predicted to reach 21.1% by 2050 (United Nations 2013). However, 8 out of 10 older adults currently report at least 1 chronic disease (Center for Technology and Aging 2009). As such, there is an urgent need to understand the mechanisms underpinning the ageing process, not only to increase life expectancy but also to improve healthspan.

Interestingly, the aged phenotype shares several metabolic similarities with that of circulatory glucocorticoid (GC) excess (Cushing’s syndrome), including insulin resistance, type 2 diabetes mellitus, central obesity, hypertension, myopathy, osteoporosis, and skin thinning (Fardet *et al.* 2007, Barzilai *et al.* 2012). As such, it is plausible that GCs somehow play a central role in the ageing process.

The active GC in rodents, corticosterone (equivalent of cortisol in humans), is synthesised in the adrenal glands, with secretion tightly controlled by the hypothalamic–pituitary–adrenal (HPA) axis. Studies examining age-associated changes in the HPA axis have had mixed results, although some demonstrate blunting of cortisol diurnal secretion along with increases in mean levels (Dodt *et al.* 1994, Van Cauter *et al.* 1996). GC availability and action depend not only upon circulating levels but also on tissue-specific intracellular metabolism by 11β-hydroxysteroid dehydrogenases (11β-HSDs) (Gathercole *et al.* 2013). Key metabolic tissues including the liver, adipose tissue, and skeletal muscle express 11β-hydroxysteroid dehydrogenase type 1 (11β-HSD1), which converts inactive 11-dehydrocorticosterone (11DHC) to active corticosterone (cortisone and cortisol in humans, respectively) (Morgan *et al.* 2009).

Conversely, 11β-hydroxysteroid dehydrogenase type 2 (11β-HSD2) is predominately expressed in the kidney, colon, and salivary gland and catalyses the inactivation of corticosterone to 11DHC. This not only protects the mineralocorticoid receptor from occupancy by cortisol but also provides a substrate for reactivation by 11β-HSD1 in peripheral tissues (Ferrari & Krozowski 2000).

Interestingly, an age-related increased 11β-HSD1 activity has been identified in human primary osteoblasts, skin dermal fibroblasts, the hippocampus, and brown adipose tissue (BAT). This, in turn, may result in increased local GC availability in these tissues, potentially contributing to age-related osteoporosis, skin thinning, cognitive decline, and impaired metabolic BAT function, respectively (Cooper *et al.* 2002, Holmes *et al.* 2010, Tiganescu *et al.* 2011, Doig *et al.* 2017). However, the role of local GC metabolism by 11β-HSD1 in the liver, adipose tissue, and skeletal muscle, in the context of ageing, is unclear.

In the present study, we use transgenic animal models to test the hypothesis that age-related changes in body composition (central adiposity and decreased muscle mass) and resulting chronic disease (type 2 diabetes, sarcopenia, and hypertension) are caused by excessive GCs as a consequence of increased circulatory levels and/or increased local tissue generation by 11β-HSD1. This was addressed by characterising the impact of ageing on circulating GC levels, pre-receptor GC metabolism, adiposity, muscle strength and mass, blood pressure, and insulin tolerance in both wildtype (WT) and transgenic male mice with a global deletion of 11β-HSD1.

## Design and methods

### Mouse ageing protocol

All animal procedures were approved by the Animals (Scientific Procedures) Act 1986 of the United Kingdom (Project Licences: 30/2764), as well as the University of Birmingham’s Animal Welfare and Ethical Review Body. Male C57BL/6 WT and 11β-HSD1 knockout (KO) (Semjonous *et al.* 2011) mice were maintained on a normal chow diet (composition in kcal: fat: 12%, carbohydrate: 60% protein: 28%) for 24 months (‘aged’) and culled in parallel with 12-week-old chow-fed animals of the same genotype (‘young’). In a separate cohort, male C57BL/6 WT and 11β-HSD1 KO mice were maintained on normal chow for 18 months prior to being transferred to a 45% high-fat (HF) diet (composition in kcal: fat: 45%, carbohydrate: 35% protein: 20%) (Research Diets, New Brunswick, NJ, USA). Young controls were transferred onto the HF diet immediately following weaning. Both young and aged animals were maintained on this diet for 12 weeks prior to euthanasia. At the end of the experiment, animals were culled by cervical dislocation and tissues excised, weighed, and snap-frozen using liquid nitrogen for later analyses. Tissue weights were normalised to kidney weight (rather than body weight) due to the marked obesity of the aged HF-fed 11β-HSD1 KO mice in comparison to the other groups. See Supplementary Figure 1A (see section on supplementary materials given at the end of this article) for the kidney weight data used for normalisation.

### Metabolic parameters

Glucose tolerance was assessed 3 weeks prior to euthanasia by fasting mice for 16 h before blood glucose was measured from tail vein nicks using a glucometer (Accu-Chek, Roche) at 0, 15, 30, 60, 90, and 120 min post glucose i.p. injection (2 g/kg). A blood sample was taken at baseline for insulin determination, measured using the Ultra Sensitive Mouse Insulin ELISA kit (Crystal Chem, Inc., Downers Grove, IL, USA). A homeostatic model assessment of insulin resistance (HOMA-IR) score was calculated with the formula: fasting plasma glucose (mM) × fasting serum insulin (μU/L) divided by 22.5. Low HOMA-IR values indicate high insulin sensitivity, whereas high HOMA-IR values indicate low insulin sensitivity (insulin resistance). Grip strength was assessed 2 weeks prior to euthanasia using a digital grip-strength metre (Linton Instrumentation, Leeds, UK). Each mouse underwent four repeat readings before results were averaged and normalized to body weight.

### Analysis of urinary steroid metabolites

Urine was collected daily from each mouse on filter paper, at 09:00  h for 2 weeks prior to euthanasia. Steroid extraction and analysis were carried out using gas chromatography-mass spectrometry (GC/MS) as previously described (Lavery *et al.* 2006).

### Cortisone acetate challenge and cortisol generation after gavage

Six weeks prior to euthanasia, animals were administered a cortisone acetate suspension in water (0.25 mg in 200 μL) by gavage. After 20 min, a 150 μL blood sample was taken by tail venesection, and cortisol generated was assessed by GC/MS analysis as described previously (Lavery *et al.* 2012).

### Morning serum corticosterone quantification

Blood samples were taken at 08:00  h by tail venesection and immediately centrifuged at 1000 ***g*** in heparin-coated tubes. Serum was transferred to cryotubes and snap-frozen. Corticosterone levels were measured by ELISA according to the manufacturer’s instructions (Abcam).

### Blood pressure assessment

Blood pressure and pulse rate were measured using tail-cuff plethysmography according to the manufacturer’s instructions (BP-2000 Blood Pressure Analysis System, Visitech Systems, Apex, NC, USA). Briefly, mice were restrained and tail cuffs with pneumatic pulse sensors attached to tails. Mice were habituated to this procedure for 10 days before measurements were recorded. Results presented are averaged systolic blood pressure measurements taken over 10 days 4 weeks prior to euthanasia.

### Hepatic triglyceride quantification

Hepatic triglyceride content was measured using a colourimetric assay according to the manufacturer’s instructions (BioVision). Samples were prepared by homogenizing 100 mg of liver tissue in 1 mL of 5% Nonidet P-40 in water, samples were heated to 80–100°C in a water bath for 2–5 min and then allowed to cool to room temperature. The heating step was repeated to solubilize all triglycerides, upon which samples were centrifuged for 2 min to remove any insoluble material. Samples were diluted 10-fold with dH_2_O before being subjected to a triacylglycerol (TAG) assay.

### 11β-HSD1 and 11β-HSD2 enzyme assays in tissue explants

11β-HSD1 oxo-reductase activity (11DHC to corticosterone) was assessed by incubating freshly dissected tissue explants with 100 nm 11DHC diluted in 1 mL media (in glass tubes) with tracer amounts of [^3^H]-11DHC (synthesized in-house (Bujalska *et al.* 2002)) at 37°C for 2 h. 11β-HSD2 dehydrogenase activity (corticosterone to 11DHC) was assessed by incubating freshly dissected tissue explants with 100 nm corticosterone diluted in 1 mL media (in glass tubes) with tracer amounts of [^3^H]-corticosterone (PerkinElmer) at 37°C for 2 h. Following incubation, steroids were extracted from the medium with dichloromethane, separated by thin-layer chromatography with chloroform/ethanol (92:8), and the fractional conversion of the steroids was calculated by scanning analysis with a Bioscan 2000 radioimaging detector (Bioscan, Washington, DC, USA). Percentage conversion was normalized to tissue weight.

### RNA extraction and real-time PCR

Total RNA was extracted from tissue using the Tri-Reagent system. RNA integrity was assessed by electrophoresis on 1% agarose gel. Concentration was determined spectrophotometrically at OD_260_. In a 50 μL volume, 500 ng of total RNA was incubated with 250 μM random hexamers, 500 μM dNTPs, 20 U RNase inhibitor, 63 U Multiscribe reverse transcriptase, 5.5 mM MgCl, and 1× reaction buffer. The RT reaction was carried out at 25°C for 10 min, 48°C for 30 min before the reaction was terminated by heating to 95°C for 5 min. mRNA levels were determined using an ABI 7500 sequence detection system (Applied Biosystems). Reactions were performed in singleplex in 10 μL volumes on 96-well plates in reaction buffer containing 2× TaqMan Universal PCR Master Mix (Applied Biosystems). Primers and probes were supplied by Applied Biosystems as premade ‘assay on demands’. The specific assays used include: InsR (Mm01211875_m1), insulin receptor substrate-1 (IRS1) (Mm01278327_m1), phosphatidyl inositol-3 kinase (PI3K) (p85) (Mm01282781_m1), PI3K (p110) (Mm00435673_m1), AKT1 (Mm01331626_m1), AKT2 (Mm00545827_m1), PDK1 (Mm00554300_m1), AKT substrate-160 (AS160) (Mm00557659_m1), glucose transporter type 4 (GLUT4) (Mm00436615_m1), PTP1B (Mm00448427_m1), SHP2 (Mm00448434_m1), PP2A (Mm00479816_m1), and PTEN (Mm00477208_m1). All reactions were normalized against the housekeeping gene 18S rRNA, which was unaffected by genotype, age, and diet. All target genes were labelled with FAM, and the reference gene with VIC. The reaction conditions were as follows: 95°C for 10 min, then 40 cycles of 95°C for 15 s and 60°C for 1 min. Data were obtained as Ct values (Ct = cycle number at which logarithmic PCR plots cross a calculated threshold line) and used to determine ∆Ct values (∆Ct = (Ct of the target gene) − (Ct of the reference gene)). Data were expressed as arbitrary units using the following transformation (arbitrary units (AU) = 1000 × (2^−ΔCt^)).

### Statistical analysis

Statistical comparisons were performed using SPSS (IBM). Data are presented as mean ± s.e.m. with statistical significance defined as *P* < 0.05. Shapiro–Wilk normality test was used to confirm Gaussian distribution prior to performing parametric tests. See figure legends for details of which statistical tests were used. Two-way ANOVA followed by Bonferroni’s multiple comparison *post hoc* test was used to compare the effect of age and genotype. Statistical analysis on real-time PCR data was performed on ∆Ct values and not A.U.

## Results

### Ageing in WT mice leads to increased circulating corticosterone levels and dysregulated pre-receptor GC metabolism

Since the ageing phenotype shares several similarities with that of Cushing’s syndrome, we assessed whether the ageing process is associated with an increase in GC exposure (either systemically, through increased circulating levels, or locally within key metabolic tissues, through increased activity of 11β-HSD1). This was investigated using both young (12 weeks) and old (24 months) male chow-fed WT and 11β-HSD1 KO mice on the C57BL/6 background.

**Figure 1:**
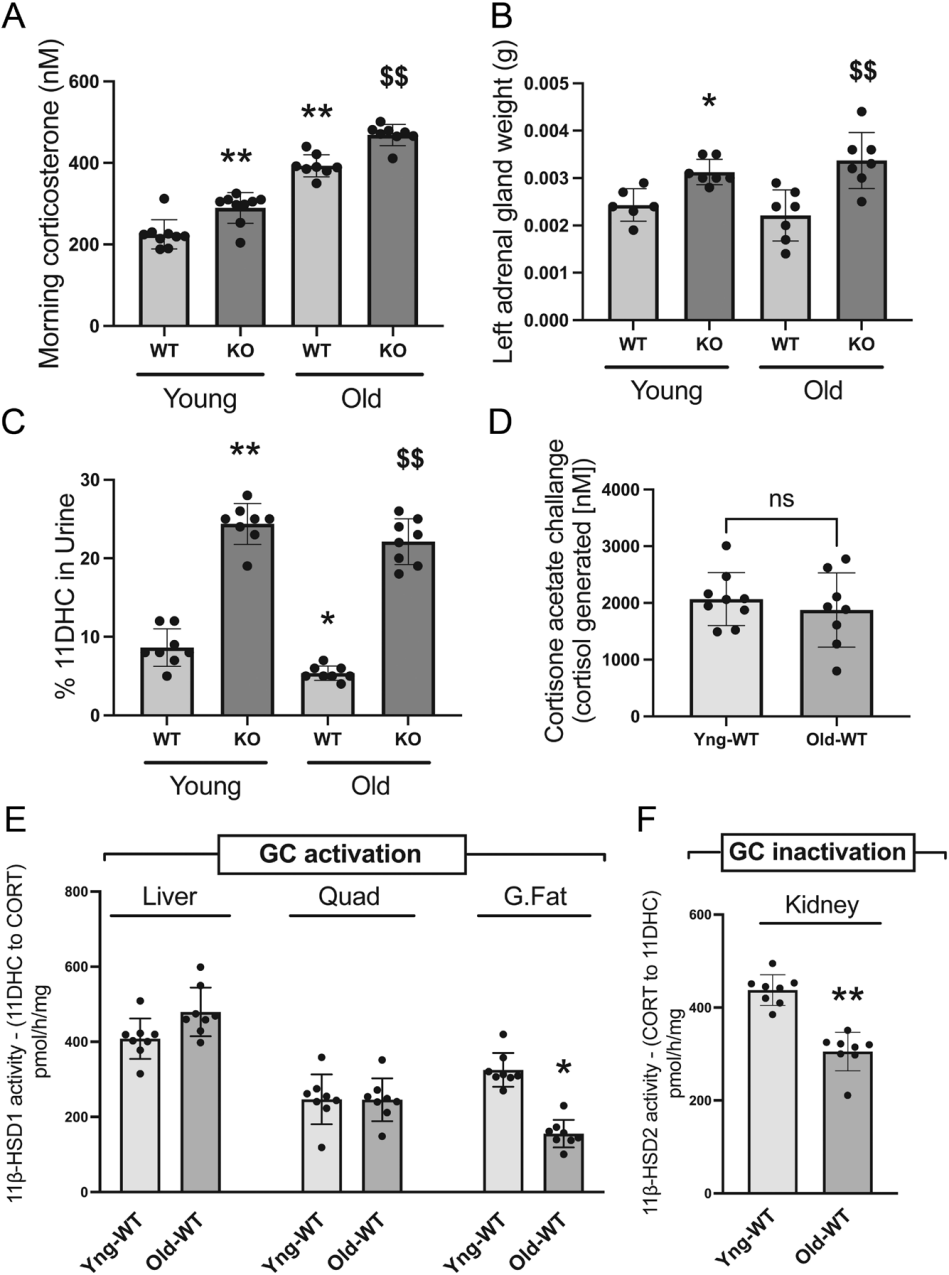
Chow-fed male C57BL/6 wildtype (WT) and 11β-HSD1 knockout (KO) mice were aged 24 months (‘Old’) and assessed in parallel with young controls animals (‘Young’, 12 weeks) of the same genotypes. Morning serum corticosterone levels were increased in WT mice with age (A). KO animals displayed adrenal hypertrophy (B), in agreement with a compensatory increased hypothalamic–pituitary–adrenal axis activation, and elevated morning serum corticosterone levels. 11-dehydrocorticosterone (11DHC) excreted in the urine of aged WT mice was decreased with age and increased in the KOs compared to the WTs (C). A cortisone acetate challenge revealed no difference in hepatic 11β-HSD1 activity in WT mice with age (D). Similarly, there were no age-related changes in 11β-HSD1 oxo-reductase activity (11DHC to corticosterone) in the liver or skeletal muscle tissue explants from WT mice, whereas decreased oxo-reductase activity was detected in gonadal adipose tissue explants (E). 11β-HSD2 dehydrogenase activity (corticosterone to 11DHC) in kidney explants from WT mice was decreased with age (D). Statistical analysis for A–C was done using two-way ANOVAs followed by Bonferroni’s multiple comparison *post hoc* test to compare the effects of age and genotype, whereas unpaired Student’s *t*-tests were used for D–F to compare the effects of age. An *n* of 8–9 animals was used in each group. (*P < 0.05; **P < 0.01 vs young WT; ^$$^*P* < 0.01 vs old WT; ns = not significant).

Consistent with a compensatory activation of the HPA axis, increased circulating morning corticosterone levels were identified in both young and old 11β-HSD1 KO mice (Fig. 1A), paralleled by adrenal hyperplasia in these animals (Fig. 1B). Furthermore, the KOs had elevated urinary excretion of 11DHC, confirming their attenuated ability to activate 11DHC to corticosterone in peripheral tissues (Fig. 1C). We also identified a 1.7-fold increase in plasma corticosterone levels in WT mice with age (Fig. 1A). In addition, a small but significant decrease in the percentage of urinary 11DHC was identified in aged WT mice (Fig. 1C), suggestive of an age-related increase in peripheral GC activation. However, hepatic 11β-HSD1 activity *in vivo*, as measured by a cortisone acetate challenge, was unchanged with age (Fig. 1D), as was 11β-HSD1 mRNA expression and oxo-reductase activity (11DHC to corticosterone) in liver explants (Fig. 1E and Table 1). Additionally, no age-related changes in the mRNA expression or oxo-reductase activity of 11β-HSD1 were identified in skeletal muscle, s.c. adipose tissue, or mesenteric adipose tissue of WT mice (Fig. 1E and Table 1). Despite this, a significant decrease in 11β-HSD1 oxo-reductase activity was identified in gonadal adipose tissue from WT mice with age (Fig. 1E).

**Table 1:** Analysis of genes involved in mediating GC metabolism and action in key metabolic tissues from both young (12 weeks) and old (24 months) wildtype and 11β-HSD1 knockout (KO) mice.

	Gene	mRNA expression (arbitrary units) ± SE					
		Young		Old		Fold change in expression with age	
		Wildtype	11β-HSD1 KO	Wildtype	11β-HSD1 KO	Wildtype	11β-HSD1 KO
Liver	11β-HSD1	0.0368 ± 0.0028	N.D	0.0381 ± 0.0034	N.D	1.04	
	GRα	0.0033 ± 0.0002	0.0039 ± 0.0001	0.0038 ± 0.0002	0.0033 ± 0.0005	1.15	0.85
Gon. adipose	11β-HSD1	0.00804 ± 0.0023	N.D	0.0011 ± 0.0003 ^¶¶^	N.D	0.14^¶¶^	
	GRα	0.0117 ± 0.0028	0.0085 ± 0.0022	0.0049 ± 0.0011^¶^	0.0069 ± 0.0015 ^¶^	0.42^¶^	0.81
Sub. adipose	11β-HSD1	0.0073 ± 0.0009	N.D	0.0070 ± 0.0011	N.D	0.96	
	GRα	0.0108 ± 0.0052	0.0121 ± 0.0022	0.0082 ± 0.0014	0.0112 ± 0.0040	0.76	0.93
Mes. adipose	11β-HSD1	0.0123 ± 0.0033	N.D	0.0098 ± 0.0024	N.D	0.80	
	GRα	0.0297 ± 0.007	0.0334 ± 0.005	0.0312 ± 0.0023	0.0360 ± 0.0063	1.05	1.08
Quad. muscle	11β-HSD1	0.00032 ± 0.00008	N.D	0.00034 ± 0.00014	N.D	1.06	
	GRα	0.0036 ± 0.0006	0.0026 ± 0.0003	0.0013 ± 0.0001	0.0024 ± 0.0009	0.36	0.92
Soleus muscle	11β-HSD1	0.00021 ± 0.00002	N.D	0.00018 ± 0.00003	N.D	0.86	
	GRα	0.00066 ± 0.00008	0.00095 ± 0.00021	0.00061 ± 0.00007	0.00044 ± 0.00012	0.92	0.46
Kidney	11β-HSD1	2.64 × 10−7 ± 1.35 × 10^-7^	N.D	2.01 × 10−7 ± 4.14 × 10^-8^	N.D	0.76	
	11β-HSD2	0.0074 ± 0.0008	0.0082 ± 0.0009	0.0049 ± 0.0008^¶^	0.0052 ± 0.0012	0.66^¶^	0.63

Statistical analysis was done using unpaired Student’s *t*-tests for comparing 11β-HSD1 expression between young and old WTs, whereas two-way ANOVA followed by Bonferroni’s multiple comparison post hoc test was used to compare the effects of age and genotype on GR expression. An *n* of 7–9 animals was used in each group.

Gon. Fat, gonadal adipose tissue; Mes. Fat, mesenteric adipose tissue; N.D, not detected; Sub. Fat, s.c. adipose tissue; Quads, quadriceps muscles.

^¶^*P* < 0.05 vs young WT; ^¶¶^*P* < 0.01 vs young WT.

The dehydrogenase activity (corticosterone to 11DHC) and mRNA expression of 11β-HSD2 was decreased in the kidneys of WT mice with age (Fig. 1F and Table 1). This suggests that the ability of the kidneys to inactivate circulating corticosterone declines in an age-dependent manner.

The expression of glucocorticoid receptor-α (GRα) mRNA was unchanged with age in the liver, quadriceps muscle, soleus muscle, s.c. adipose tissue, and mesenteric adipose tissue of WT mice. However, an age-associated decrease was identified in gonadal adipose tissue of WT animals, which may function as a protective mechanism to limit the adverse detrimental effects of local GC regeneration in this tissue (Table 1).

### Aged 11β-HSD1 KO mice have a metabolically favourable fat distribution, despite greater overall obesity

**Figure 2:**
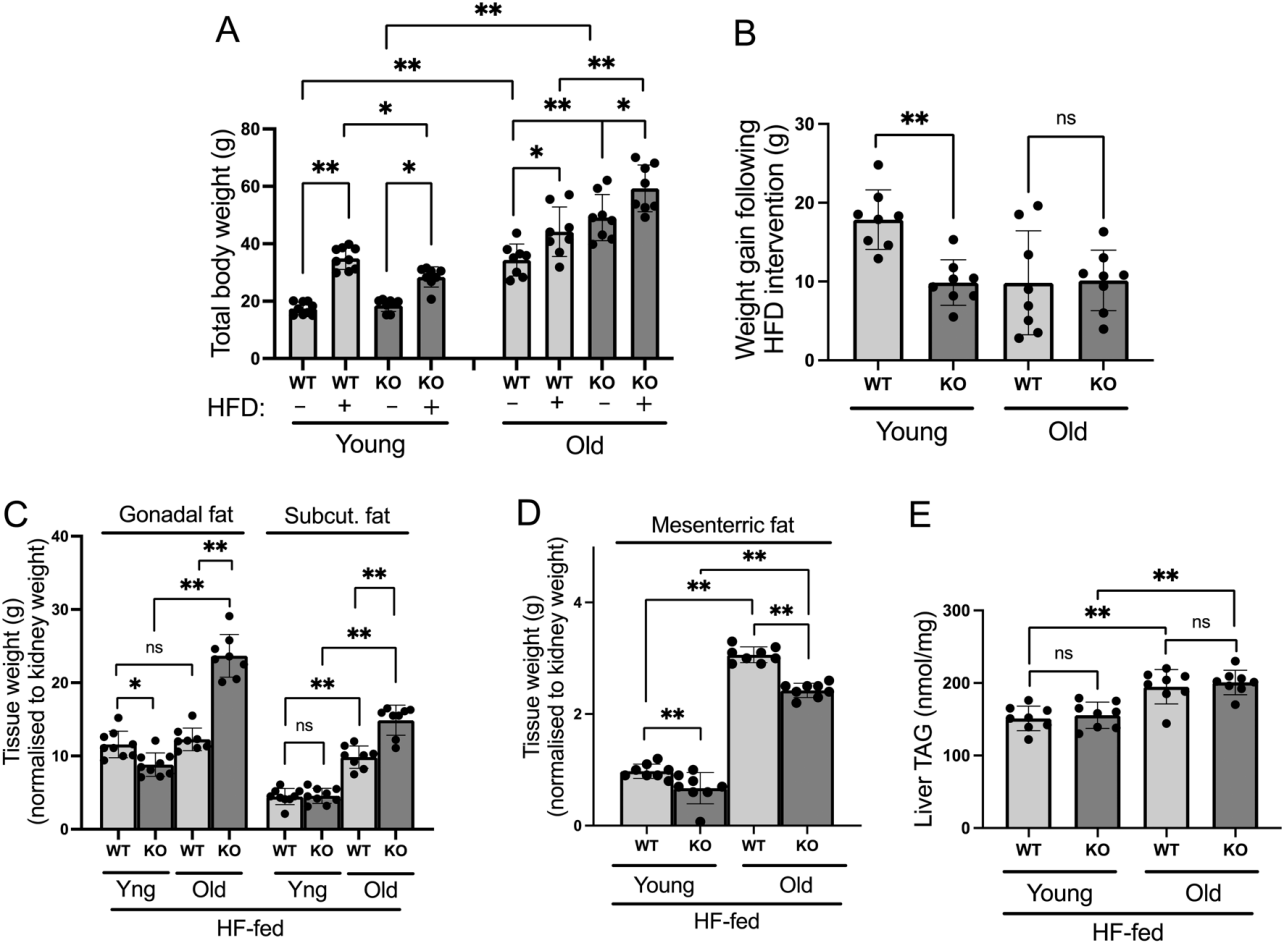
Total body weight analysis of wildtype (WT) and 11β-HSD1 knockout (KO) mice fed on chow with or without a 12-week 45% high-fat (HF) diet intervention (A). Young KO mice were partially protected from diet-induced obesity, whereas this partial protection was lost with age (B). In agreement with greater overall obesity, the aged HF-fed 11β-HSD1 KO mice had increased gonadal and s.c. adiposity in comparison to aged HF-fed WTs (C). However, the aged HF-fed 11β-HSD1 KO mice were partially protected from increased fat accumulation in the metabolically detrimental mesenteric adipose depot in comparison to aged HF-fed WTs (D). Hepatic triglyceride (TAG) content was increased with age but unaffected by genotype in HF-fed mice (E). Statistical analysis was done using three-way ANOVA followed by Bonferroni’s multiple comparison *post hoc* test for A to compare the effect of age, genotypes, and diet, whereas two-way ANOVA followed by Bonferroni’s multiple comparison *post hoc* test was used for B–E to compare the effect of age and genotypes. An *n* of 7–9 animals was used in each group. (*P < 0.05, **P < 0.001; ns = not significant, HFD, high-fat diet).

Since the ageing process is associated with increased adiposity, we assessed the fat phenotype of both young and old WT and 11β-HSD1 KO male mice. In agreement with previously published reports (Morton *et al.* 2004), we have shown that young 11β-HSD1 KO mice are partially protected from diet-induced obesity when fed on a 45% HF diet for 12-weeks – gaining ≈8 g less than the young WTs during this time (Fig. 2A and B). Surprisingly, this partial protection was lost with age, with the aged 11β-HSD1 KOs gaining a similar amount of weight as the aged WTs during the 12-week HF diet intervention. Furthermore, the aged 11β-HSD1 KOs were more obese than their aged WT controls, and this trend held true for both the chow and 12-week HF-fed animals. In agreement with greater overall obesity, the old HF-fed 11β-HSD1 KO mice had increased gonadal and s.c. adiposity in comparison to aged HF-fed WTs (Fig. 2C). By contrast, the increased mesenteric adiposity observed in the HF-fed WTs with age was blunted in the aged HF-fed 11β-HSD1 KO animals (Fig. 2D), implying that the latter store lipids in a more metabolically favourable manner with age despite greater overall obesity. Although the old chow-fed 11β-HSD1 KO mice also had greater s.c. adiposity in comparison to aged chow-fed WTs (Supplementary Fig. 1A), there was no protection for increased mesenteric adiposity with age in these animals (Supplementary Fig. 1B). Assessment of the hepatic phenotype in these animals revealed an increase in hepatic triglyceride content with age but genotype was without effect (Fig. 2E).

### High fat-fed 11β-HSD1 KO mice are protected from age-related insulin resistance

**Figure 3:**
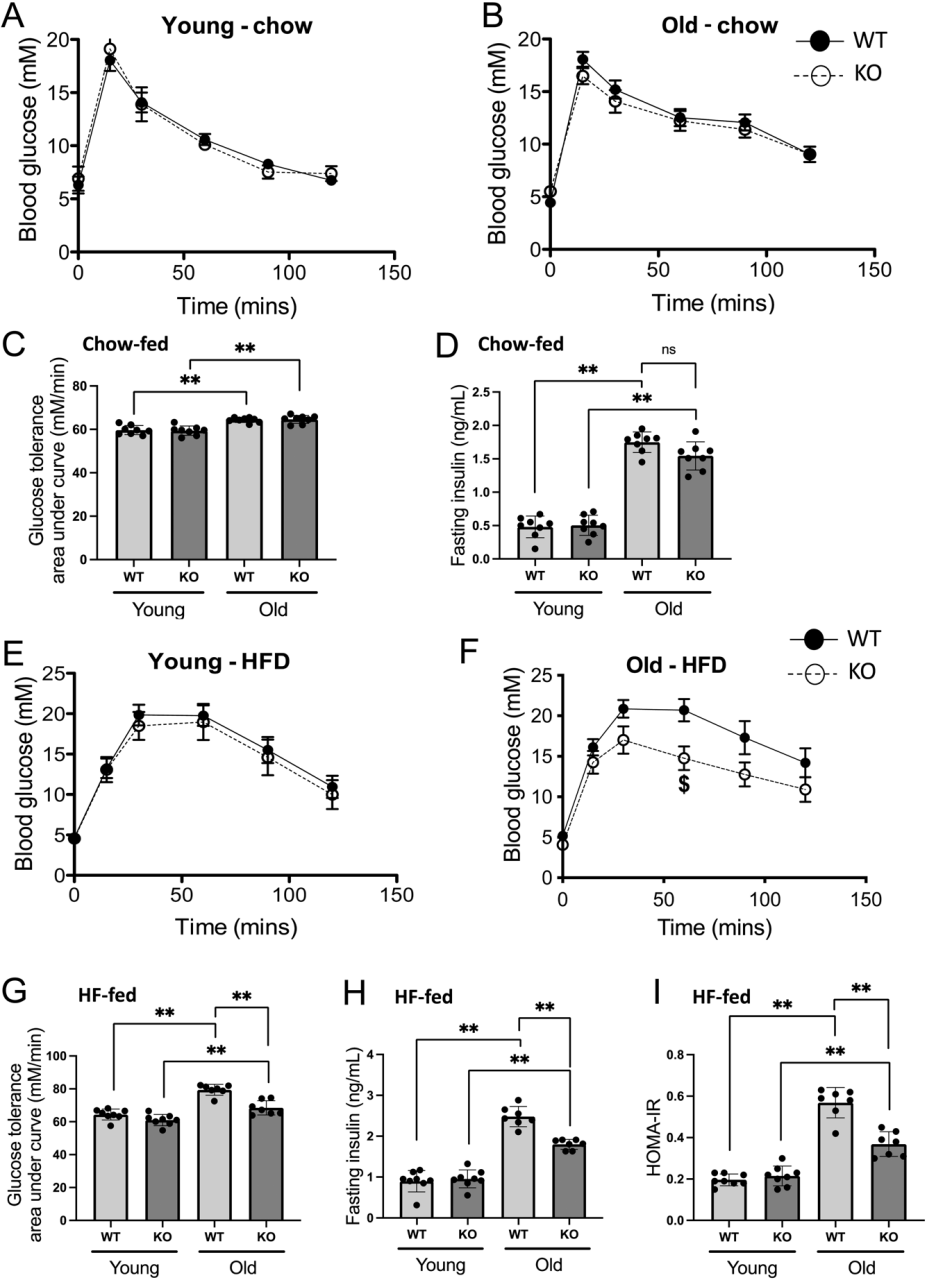
No difference in glucose tolerance was observed between chow-fed wildtype (WT, solid line) and 11β-HSD1 knockout (KO, hashed line) mice (A and B). This was confirmed from the calculated area under curves for glucose tolerance data (C). Fasting insulin levels were increased with age in both chow-fed WT and 11β-HSD1 KO mice (D). No difference in glucose tolerance was observed between young WT (solid line) and young 11β-HSD1 KO (hashed line) mice fed on a 45% high-fat (HF) diet for 12 weeks (E). Aged HF-fed WT mice were glucose intolerant in comparison to young HF-fed controls, whereas aged HF-fed 11β-HSD1 KOs had improved glucose tolerance compared to the aged HF-fed WTs (F). This was confirmed from the calculated area under curves for glucose tolerance data (G). Fasting serum insulin levels (H) and calculated HOMA-IR index (which inversely correlated with insulin sensitivity) (I) were both higher in aged HF-fed WT mice compared to the young WT controls, whereas these parameters were lower in the aged HF-fed KOs compared to aged HF-fed WTs. Statistical analysis was done using two-way ANOVA followed by Bonferroni’s multiple comparison *post hoc* test to compare the effects of age and genotype. An *n* of 7–9 animals was used in each group. (**P < 0.001, ^$^ *P* < 0.05 vs old-WT; HFD, high-fat diet; ns, not significant).

Since the ageing process is associated with reduced insulin sensitivity and type 2 diabetes mellitus, we assessed aspects of glucose metabolism in both young and old male WT and 11β-HSD1 KO mice. Since the C57BL/6 genetic background are susceptible to developing features of metabolic syndrome (including insulin resistance), we assessed glucose control in not only chow-fed mice but also animals fed on a 45% HF diet for 12 weeks prior to euthanasia. In the chow-fed mice, although ageing was associated with a worsening in glucose tolerance, genotype had no effect (Fig. 3A, B and C). Similarly, an age-related increase in fasting insulin levels was observed in both chow-fed WT and 11β-HSD1 KO animals (Fig. 3D), and although the latter appeared less hyperinsulinemic compared to the aged WTs, this did not reach statistical significance.

Both the aged HF-fed WT and KO mice were more glucose intolerant (Fig. 3E, F and G), had elevated fasting insulin levels (Fig. 3H), and had a worse HOMA-IR index (Fig. 3I) in comparison to the young HF-fed animals of the same genotype. These findings are consistent with the onset of insulin resistance in both WT and KO mice with age. However, the aged HF-fed KOs had improved glucose tolerance (Fig. 3E, F and G), lower fasting insulin levels (Fig. 3H), and a superior HOMA-IR index (Fig. 3I) in comparison to aged HF-fed WTs.

Gene expression analysis of key components of the insulin signalling cascade in quadriceps muscle of HF-fed mice is shown in Table 2. Corroborating the glucose tolerance data (Fig. 3E, F, G, H and I), we have identified several genes dysregulated with age in HF-fed WT mice. These include a decrease in the mRNA expression of IRS1, altered subunit stoichiometry of PI3K (increased expression of the regulatory subunit (p85) with respect to the catalytic subunit (p110)), increased AS160 expression, and increased expression of the GLUT4 (Table 2). Consistent with their protection from age-related insulin resistance, aged HF-fed 11β-HSD1 KO mice had similar mRNA expression levels of both IRS1 and PI3K-p85 to that of young HF-fed controls (Table 2). However, both AS160 and GLUT4 expression remained elevated in the aged HF-fed KO mice suggesting insulin-mediated glucose uptake is still impaired in these animals.

**Table 2:** Gene expression analysis of key components of the insulin signalling cascade in quadriceps muscles from both young (12 weeks) and old (24 months) HF-fed wildtype and 11β-HSD1 knockout (KO) mice.

Gene	mRNA expression (arbitrary units) ± s.e.					
	Young		Old		Fold change in expression with age	
	Wildtype	11β-HSD1 KO	Wildtype	11β-HSD1 KO	Wildtype	11β-HSD1 KO
InsR	0.01071 ± 0.0009	0.0095 ± 0.0005	0.0122 ± 0.0003	0.0115 ± 0.0008	1.14	1.21
IRS1	0.0101 ± 0.0016	0.0091 ± 0.0012	0.0054 ± 0.0010^¶^	0.0083 ± 0.0009♯	0.53^¶^	0.91
PI3K (p85)	0.0081 ± 0.0012	0.0080 ± 0.0031	0.0182 ± 0.0031^¶¶^	0.0101 ± 0.0020♯♯	2.25^¶¶^	1.26
PI3K (p110)	0.0011 ± 0.0002	0.0011 ± 0.0003	0.0010 ± 0.0002	0.0011 ± 0.0003	0.91	1.00
AKT1	0.0071 ± 0.0002	0.0068 ± 0.0002	0.0079 ± 0.0003	0.0076 ± 0.0004	1.11	1.12
AKT2	0.0033 ± 0.0006	0.0036 ± 0.0007	0.0030 ± 0.0006	0.0035 ± 0.0005	0.91	0.97
PDK1	0.0081 ± 0.0010	0.0080 ± 0.0008	0.0084 ± 0.0012	0.0089 ± 0.0007	1.04	1.11
AS160	0.0036 ± 0.0010	0.0039 ± 0.0012	0.0072 ± 0.0011^¶^	0.0063 ± 0.0009^¶^	2.00^¶^	1.62^¶^
GLUT4	0.0569 ± 0.0091	0.0523 ± 0.0112	0.0921 ± 0.011^¶¶^	0.0929 ± 0.0099^¶^	1.62^¶¶^	1.78
PTP1b	0.0022 ± 0.0002	0.0008 ± 0.0001^¶¶^	0.0024 ± 0.0003	0.0007 ± 0.0001♯♯	1.09	0.88
SHP2	0.0115 ± 0.0042	0.0114 ± 0.0066	0.0100 ± 0.0031	0.0093 ± 0.0054	0.87	0.82
PP2A	0.0265 ± 0.0015	0.0127 ± 0.0021^¶^	0.0262 ± 0.0025	0.0177 ± 0.0032♯	0.99	1.39
PTEN	0.0101 ± 0.0063	0.0133 ± 0.0053	0.0142 ± 0.0059	0.0144 ± 0.0072	1.41	1.08

Statistical analysis was done using two-way ANOVA followed by Bonferroni’s multiple comparison *post hoc* test to compare the effects of age and genotype. An *n* of 7–9 animals was used in each group.

^¶^*P* < 0.05; ^¶¶^*P* < 0.01 vs young WT; ^♯^*P* < 0.05, ^♯♯^*P* < 0.01vs old WT.

**Figure 4:**
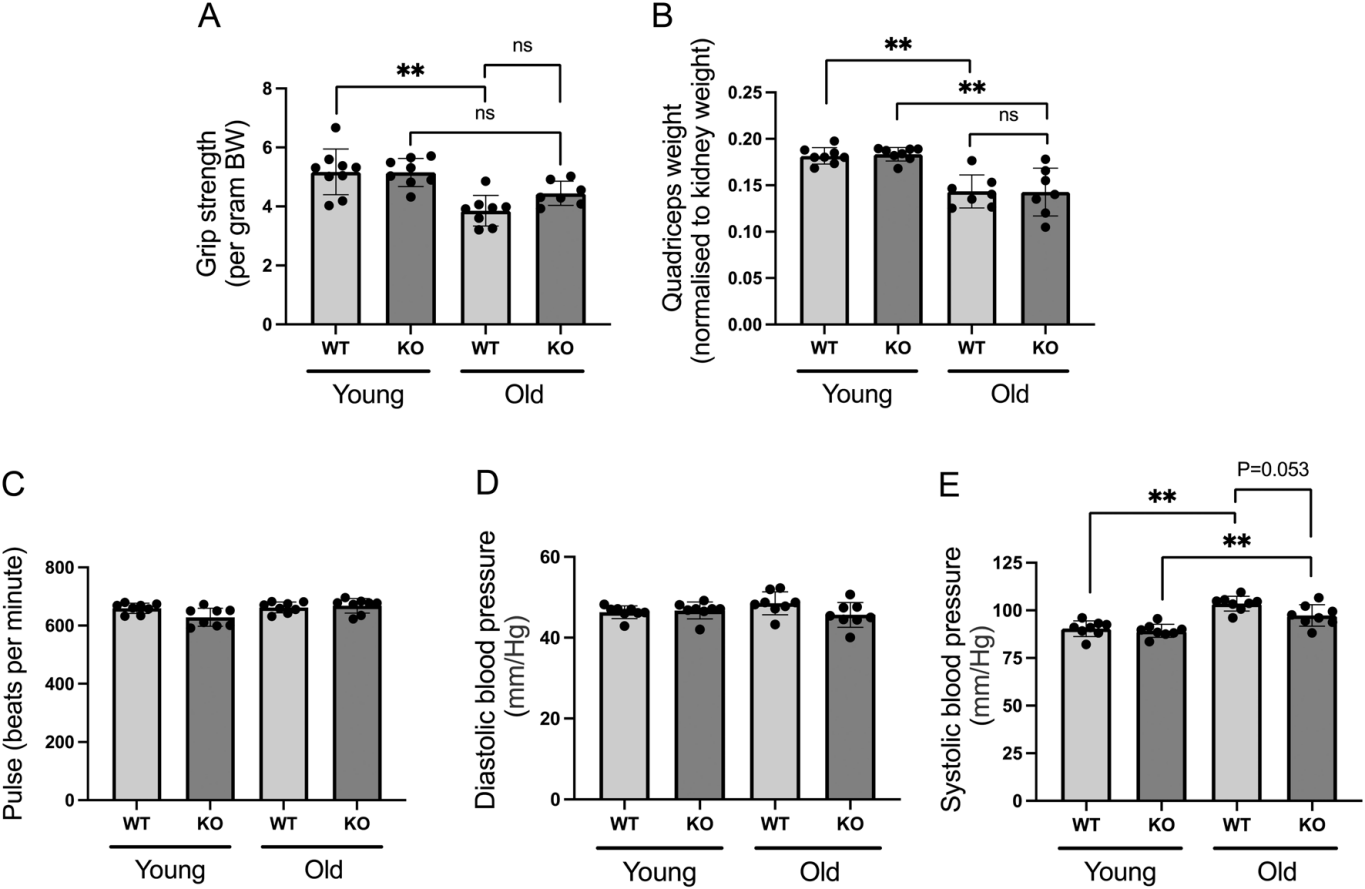
Both grip strength (A) and quadriceps muscle bed weights (B) were decreased with age in both chow-fed WT and KO mice. Pulse rate (C) and diastolic blood pressure (D) were unaffected by age or genotype, whereas systolic blood pressure (E) was increased with age in WT and KO animals. Statistical analysis was done using two-way ANOVA followed by Bonferroni’s multiple comparison *post hoc* test to compare the effects of age and genotype. An *n* of 7–9 animals was used in each group. (**P < 0.001; ns, not significant).

Since the ageing process is associated with a gradual reduction in both muscle mass and strength, we assessed the muscle phenotype as part of the present study. Consistent with the onset of sarcopenia, WT mice displayed reduced grip strength with age, paralleled by loss of mixed fibre muscle bed mass (quadriceps). However, no protection from these age-related changes was evident in 11β-HSD1 KO mice (Fig. 4A and B).

Blood pressure is well established to increase with age. As such, this parameter was assessed using pneumatic tail cuffs. Although no difference in pulse rate (Fig. 4C) or diastolic blood pressure (Fig. 4D) was observed with age or genotype in the HF-fed animals, we did detect an age-related increase in systolic blood pressure in both HF-fed WTs and KOs (Fig. 4E). Interestingly, although this effect appeared blunted in the aged HF-fed KOs, this did not reach statistical significance (*P* = 0.053). Assessment of these blood pressure parameters in the chow-fed mice mirrored those of HF-fed animals (data not shown). For all figures in this manuscript, all outlier data points were included in the analysis.

## Discussion

Currently, the molecular mechanisms underpinning the ageing process are not completely understood. Since the ageing phenotype shares many metabolic similarities with that of circulatory GC excess, it is plausible that GCs are in some way involved in driving the aged phenotype. In the present study, we have characterised the impact of ageing on circulating GC levels, pre-receptor GC metabolism, adiposity, muscle strength and mass, blood pressure, and insulin sensitivity in both WT and transgenic male mice with a global deletion of 11β-HSD1.

In agreement with previously published rodent and human studies (Van Cauter *et al.* 1996, Ferrari *et al.* 2001, Giordano *et al.* 2005, Yau *et al.* 2007), we have shown circulating corticosterone levels to increase with age in both WT and 11β-HSD1 KO mice – likely driven by increased HPA axis activation. Ideally, this would be confirmed by measuring the serum adrenocorticotrophic hormone (ACTH) levels. However, since secretion of this hormone increases rapidly in response to stress (e.g. collecting blood samples), obtaining accurate measurements for ACTH was not possible in our hands. Despite this, others have shown a small age-related increase in the area under the curve for ACTH secretion in humans (Giordano *et al.* 2005).

Assessment of insulin sensitivity revealed that chow-fed 11β-HSD1 KO mice were not protected from the development of glucose intolerance, hyperinsulinaemia, or a worsening HOMA-IR index with age. Therefore, we ‘stressed the system’ by implementing a 12-week HF diet intervention. Importantly, the mice used in this study were bred on the C57BL/6 background, which is susceptible to developing features of the metabolic syndrome (including insulin resistance). As anticipated, aged HF-fed WTs became fully glucose intolerant, insulin resistant, and hyperinsulinemic, in comparison to young HF-fed controls, suggesting age rendered these animals more susceptible to dysregulated glucose control. Crucially, the aged HF-fed KO mice were not as glucose-intolerant, peripherally insulin resistant or hyperinsulinemic, implying that 11β-HSD1 regulates glucose homeostasis with age under HF feeding conditions. These findings were endorsed at a molecular level where the suppressed IRS1 expression and dysregulated PI3K subunit stoichiometry observed in the skeletal muscle of the aged HF-fed WT animals were not present in the aged HF-fed KOs. Importantly, GCs have been shown to directly induce insulin resistance in skeletal muscle by modulating the expression of IRS1 and PI3K in a similar manner to that reported here in WT mice with age (Morgan *et al.* 2009) – further supporting the premise that these age-dependent molecular changes are GC dependent. Despite this, there was evidence of impaired insulin-mediated glucose uptake in the skeletal muscle of the aged HF-fed KOs as indicated by elevated AS160 and GLUT4 expression. However, it should be noted that we did not evaluate total protein levels as part of the current study, as such the reported mRNA changes may not necessarily translate to total protein levels.

It is well documented that 11β-HSD1 deletion in young mice offers some protection from diet-induced obesity (Morton *et al.* 2004), and the findings presented in this study support this. However, our results also indicate that the partial protection from weight gain seen in the HF-fed young animals is lost with age – with the aged 11β-HSD1 KOs gaining a similar amount of weight during the HF diet intervention as the aged WTs. Furthermore, the aged KO animals were significantly more obese compared to their aged WT counterparts. However, post-mortem analysis revealed that, in contrast to the WTs, the aged HF-fed KOs stored lipids in a more metabolically favourable manner, despite greater overall obesity, mesenteric adipose tissue is thought to play a central role in the development of metabolic dysfunction (Yang *et al.* 2008). As such, the reduced mesenteric adiposity of the aged HF-fed KOs may contribute to the improved glucose control seen in these animals compared to the aged HF-fed WTs. This finding is consistent with the known depot-specific effects of GCs on adipose tissue (Lundgren *et al.* 2004, Hazlehurst *et al.* 2013) and may be underpinned by the relatively higher GR expression in the mesenteric compared to s.c. and gonadal depots.

Ectopic lipid deposition in peripheral tissues is a hallmark of metabolic dysfunction (Trouwborst *et al.* 2018). While we did not observe any differences in hepatic triglyceride contentment with genotype in the present study, we cannot discount the possibility that the protective metabolic phenotype observed in the aged 11β-HSD1 KO mice could be underpinned (at least in part) by reduced age-associated ectopic triglyceride accumulation in skeletal muscle or protection from an accumulation of lipotoxic lipid intermediates such as diacylglycerides and ceramides in the liver or muscle.

Consistent with the known effects of ageing in humans, we observed the onset of systolic hypertension in the aged WT and KO mice compared to their younger counterparts. This may be partly driven by the concomitant age-associated increased circulating morning corticosterone levels in these animals. Furthermore, we observed a decreased renal 11β-HSD2 expression/activity in the aged WT mice. Normally the function of 11β-HSD2 is to inactivate corticosterone locally in the kidneys for the purpose of protecting the non-selective mineralocorticoid receptor from occupancy by this metabolite, which would otherwise lead to increased sodium retention and elevated blood pressure. Thus, a reduction in its activity may also contribute to the observed systolic hypertension in these animals. Interestingly, deletion of 11β-HSD1 did not ameliorate age-associated systolic hypertension when compared to the young mice.

Our data underscore a potentially critical role for pre-receptor GC metabolism in contributing to age-associated chronic disease, but raises an important question: ‘Precisely how does 11β-HSD1 drive age-related metabolic dysfunction?’ Previous studies have shown 11β-HSD1 to increase with age in the skin, osteoblasts, hippocampus, and skeletal muscle (in females but not males), which in turn is thought to contribute to age-related skin thinning, osteoporosis, cognitive decline, and sarcopenia, respectively (Cooper *et al.* 2002, Holmes *et al.* 2010, Tiganescu *et al.* 2011, Hassan-Smith *et al.* 2015). Furthermore, our analysis of the BAT from the same cohort of mice used in the current study, which formed part of a previous publication, also reveals an age-related increase in 11β-HSD1 expression, which in turn may contribute to impaired metabolic BAT function with age (Doig *et al.* 2017). As such, an age-associated increase in local GC availability driven by increased 11β-HSD1 activity may play an important role in driving features of the ageing phenotype in certain tissues such as the skin, brain, bone, and BAT. In support of this, we identified a small but significant decrease in the percentage of urinary excretion of 11DHC in aged WT mice, suggestive of an age-related increase in peripheral GC activation. However, we did not identify age-associated increases in 11β-HSD1 expression/activity in the liver, adipose tissue, or skeletal muscle suggesting the mechanism by which 11β-HSD1 contributes to features of the ageing phenotype in these tissues is not explained by a simple increase in its expression/activity with age. Previously, we demonstrated that 11β-HSD1 governs intracellular access to circulating corticosterone in mice, and in turn plays a central role in driving the metabolic complications associated with circulatory GC excess (Tomlinson *et al.* 2002, Morgan *et al.* 2014). Mechanistically, this stems from the fact that a proportion of circulating corticosterone is inactivated by 11β-HSD2, predominantly in the kidneys, and the resultant 11DHC acts as a substrate for 11β-HSD1 and is subsequently reactivated to corticosterone within key metabolic tissues in the vicinity of the GR (Lin *et al.* 1997, Cooper *et al.* 2002). Since circulating corticosterone levels increase with age in WT mice, potentially so could the proportion inactivated to 11DHC in the kidneys. This increased substrate availability for 11β-HSD1 could, in turn, result in enhanced local corticosterone reactivation within peripheral tissues, contributing to age-associated metabolic dysfunction observed in the WT mice. This could potentially explain how 11β-HSD1 contributes to age-related metabolic dysfunction regardless of whether its expression/activity increases with age (skin, brain, bone, and BAT) or remains unchanged (liver, adipose, and skeletal muscle). However, this remains to be fully tested in this context.

A limitation of the current study is that the role of 11β-HSD1 in driving aspects of the ageing phenotype was only investigated in male mice. As such, we can discount the possibility of sexual dimorphism with respect to our findings. Indeed, there is evidence of sexually dimorphic expression of hepatic 11β-HSD1 in rats, underpinned by sex-specific changes in growth hormone secretion patterns (Low *et al.* 1994, Albiston *et al.* 1995). Furthermore, Hassan-Smith *et al.* have shown 11β-HSD1 increases with age in the skeletal muscle of women, but not men, and this increase correlates with features of the ageing phenotype including sarcopenia and insulin resistance (Hassan-Smith *et al.* 2015). In agreement, we also did not observe an age-related increase in skeletal muscle 11β-HSD1 expression/activity in aged male WT mice, nor did we see protection from age-related sarcopenia in these animals. Although, we cannot confirm whether the opposite is true in female animals. Therefore, it is important that future studies focus on both sexes to identify whether any apparent 11β-HSD1-dependent age-related changes are universal or gender-specific.

The present study raises the intriguing possibility of selectively targeting 11β-HSD1 as a novel therapeutic strategy to improve health in old age. To date, pre-clinical and clinical studies have shown selective 11β-HSD1 inhibitors to modestly improve insulin sensitivity, reduce dyslipidemia and reverse central obesity in patients with type 2 diabetes, and improve cognitive function in aged mice (Rosenstock *et al.* 2010, Feig *et al.* 2011, Sooy *et al.* 2015). Although their efficacy in ameliorating the adverse metabolic complication associated with the ageing process has not yet been fully tested, Hardy *et al.* have recently demonstrated a beneficial effect of selective 11β-HSD1 inhibition on lean mass in obese female patients with intracranial hypertension (age range: 18–55 years), highlighting potential utility for the treatment of sarcopenia (Hardy *et al.* 2021).

In conclusion, we have demonstrated that 11β-HSD1 plays a critical role in driving age-related insulin resistance and a metabolically harmful fat distribution in HF-fed, but not chow-fed male mice, underscoring the potential for the use of a selective 11β-HSD1 inhibitor to increase healthspan.

## Supplementary Material

Supplementary Figure 1

## Declaration of interest

The authors declare that there is no conflict of interest that could be perceived as prejudicing the impartiality of the research reported.

## Funding

This work has been supported by the Wellcome Trust (program grant ref. 082809) (P M S) and the ERC Advanced Research Grant (PRECORT) (P M S).
