# Supplementary figures and images for: 11β-HSD1 contributes to age-related metabolic decline in male mice
Source: J Endocrinol. 2022 Oct 7;255(3):117–29. doi: 10.1530/JOE-22-0169 (PMC9578088; doi:10.1530/JOE-22-0169)

# Supplementary figure 1

**A**

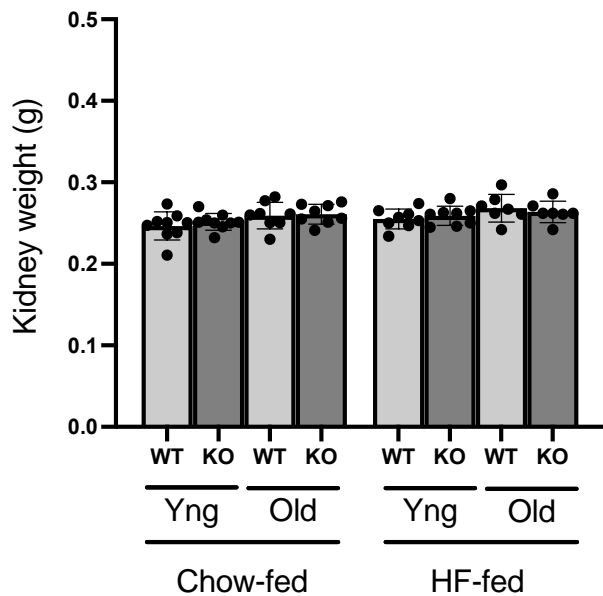

**B**

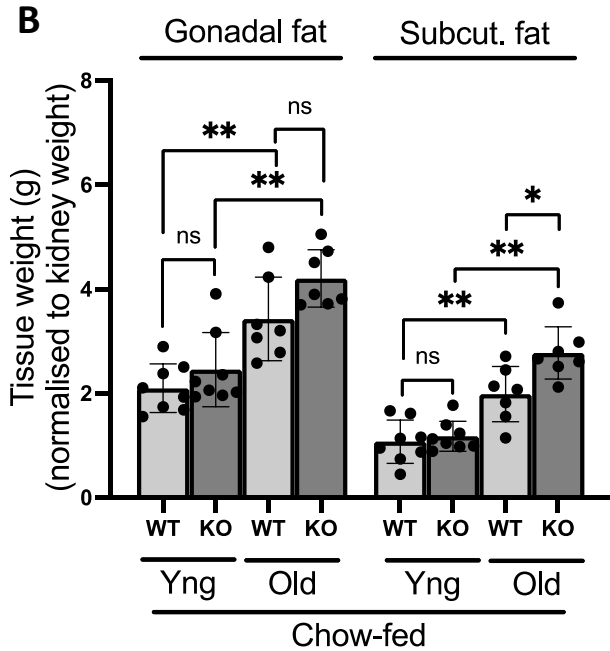

**C**

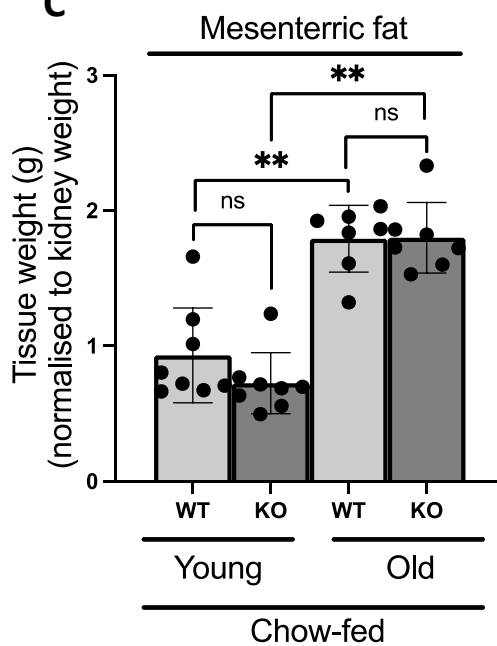

Supplement: Supplementary Figure 1 [file supplementary_figure_1.pdf]
